# Effect of intrauterine injection of human chorionic gonadotropin before fresh embryo transfer on IVF and ICSI outcomes: a meta-analysis

**DOI:** 10.1007/s00404-018-4923-1

**Published:** 2018-10-05

**Authors:** Wenhui Hou, Gaohui Shi, Bing Cai, Chenhui Ding, Junli Song, Xiubing Zhang, Yanwen Xu

**Affiliations:** Reproductive Medical Center, The First Affiliated Hospital, Sun Yat-sen University, Guangzhou, People’s Republic of China

**Keywords:** hCG, Intrauterine injection, Meta-analysis, IVF, ICSI, Fresh embryo transfer

## Abstract

**Purpose:**

This analysis was performed to evaluate the effects of intrauterine injection of human chorionic gonadotropin (hCG) before fresh embryo transfer (ET) on the outcomes of in vitro fertilization and intracytoplasmic sperm injection.

**Methods:**

Randomized controlled trials (RCTs) were identified by searching electronic databases. The outcomes of live birth, clinical pregnancy, implantation, biochemical pregnancy, ongoing pregnancy, ectopic pregnancy, and miscarriage between groups with and without hCG injections were analyzed. Summary measures were reported as risk ratios (RR) with 95% confidence intervals.

**Results:**

Six RCTs on fresh embryo transfer (ET) were included in the meta-analysis. A total of 2759 women undergoing fresh ET were enrolled (hCG group *n* = 1429; control group *n* = 1330). Intrauterine injection of hCG significantly increased rates of biochemical pregnancy (RR 1.61) and ongoing pregnancy (RR 1.58) compared to controls. However, there were no significant differences in clinical pregnancy (RR 1.11), implantation (RR 1.17), miscarriage (RR 0.91), ectopic (RR 1.65) or live birth rates (RR 1.13) between the hCG group and control group.

**Conclusion:**

The current evidence for intrauterine injection of hCG before fresh ET does not support its use in an assisted reproduction cycle.

## Introduction

Approximately one in six-to-seven couples suffer from infertility worldwide [1]. Despite advances in assisted reproductive techniques (ARTs), the pregnancy rate remains unsatisfactory [2–4]. Implantation, a critical stage of pregnancy, is a complex process, such that more than half of all pregnancy failures are caused by implantation failure [5]. Three components are considered to be essential for successful implantation, process-embryo quality, endometrial receptivity, and embryo–endometrium communication [6].

Embryo–endometrium communication is regulated by autocrine and paracrine factors of which human chorionic gonadotropin (hCG) is considered the most important [7]. Embryos begin to transcript hCG at the two-cell stage and secrete hCG before implantation [5]. During the luteal phase, the endometrial epithelial cells also produce hCG, which acts in an autocrine–juxtacrine manner, until its appearance in the serum [8]. HCG regulates implantation by different mechanisms, for example, it facilitates trophoblast invasion [9], supports trophoblast apposition and adhesion, and regulates proteins involved in implantation [10].

Licht et al. created an intrauterine micro-dialysis instrument to investigate the effects of hCG on human endometrium [11]. They found that intrauterine infusion of hCG could up-regulate vascular endothelial growth factor and matrix metalloproteinase-9, which are important for tissue remodeling, suggesting that hCG plays a crucial role in angiogenesis, vascularization, and placentation of the endometrium. Several other studies have reported that hCG can promote gene expression towards tolerance, receptivity, and implantation [12, 13].

The function of hCG in the implantation process has inspired clinicians to study the effect of intrauterine hCG administration at the time of embryo transfer on ART outcomes. Several studies have examined the role of intrauterine hCG injection before fresh embryo transfer in ART, but the results have been inconsistent [3, 5, 8, 14–16]. Therefore, we conducted a meta-analysis to investigate whether intrauterine injection of hCG before fresh embryo transfer improves IVF/ICSI outcomes.

## Materials and methods

### Literature search

Comprehensive literature searches were conducted on PubMed, Web of Science, SCOPUS, and EBSCO from the date of inception to August 2017 without restriction to regions, publication types, or languages, using the search strategy [Title/Abstract]: (“human chorionic gonadotropin” or “hCG” or “rhCG” or “recombinant hCG”) AND (“intrauterine administration” or “intrauterine injection” or “intrauterine administration” or “endometrial infusion”) AND (“assisted reproductive techniques” or “ART” or “in vitro fertilization” or “IVF” or “ICSI” or “intracytoplasmic sperm injections” or “embryo transfer” or “implantation”). There were no language restrictions on any of our searches. Two authors (Hou and Shi) independently searched the publications.

### Selection criteria

The target population was women undergoing IVF/ICSI who had an intrauterine hCG injection before fresh ET and women who had ET with no intrauterine HCG injection. Two authors (Hou and Shi) independently screened the title and abstract of each publication to exclude studies that did not correspond with the objective of this review. The same two authors appraised the remaining publications by examining the full text alone to identify RCTs suitable for inclusion. Review articles and non-prospective comparative studies were not considered. Conference abstracts and dissertations were excluded. To avoid overlapping patient data in duplicate publications, we used registry analyses to cross check publications with institutional studies and then compare them with other studies in other registries. We included only the larger or more comprehensive publication in this meta-analysis.

### Assessment of the methodological quality and data extraction

The modified Jadad scale was used to evaluate the quality of each publication [17] based on the RCTs four characteristics: randomization, blinding, allocation concealment, and reporting of participant withdrawals and drop outs. The studies were rated on a scale from 0 to 7, with a total score less than four indicating low quality and all other scores indicating high quality. Two reviewers completed the assessment and data extraction.

### Statistical analysis

RevMan version 5.3 (Cochrane Collaboration, Oxford, UK) was utilized to analyze the data. Dichotomous data in all the studies were expressed in terms of their relative risk (RR) with 95% confidence intervals; all RRs were pooled to yield an overall RR. *P* < 0.05 was considered statistically significant. Treatment outcomes were analyzed using a random effects model and statistical heterogeneity was assessed using the *I*^2^ statistic [18, 19].

## Results

### Literature search

**Fig. 1:**
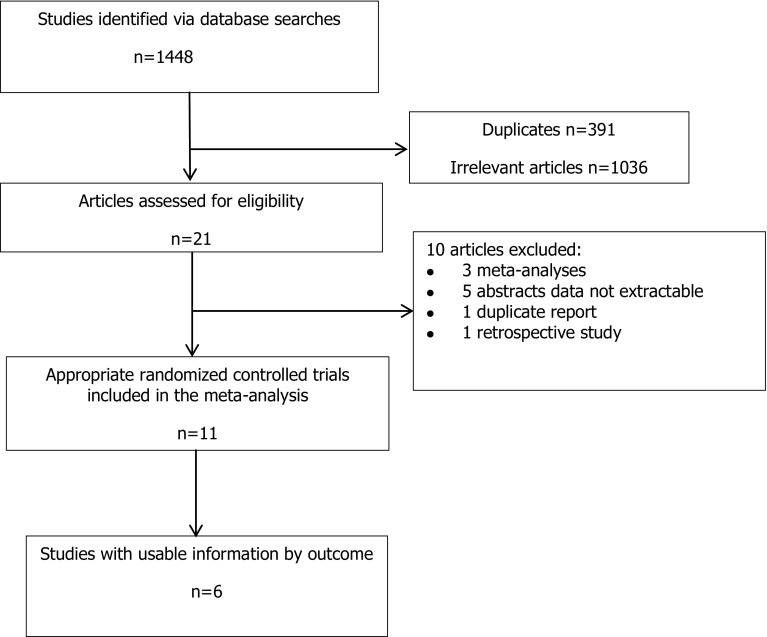
Study selection process for the meta-analysis

The literature identification and selection processes are summarized in Fig. 1. The initial screening yielded 1448 publications of which 21 were eligible. After examining the full text of the 21 articles, 10 studies were excluded (Fig. 1). Finally, six RCTs satisfied the selection criteria for a fresh ET cycle (Fig. 1). Studies were excluded if the full text was not in English, it was a retrospective study, or sufficient information about the study was not available in the English-language abstract.

### Study characteristics

**Fig. 2:**
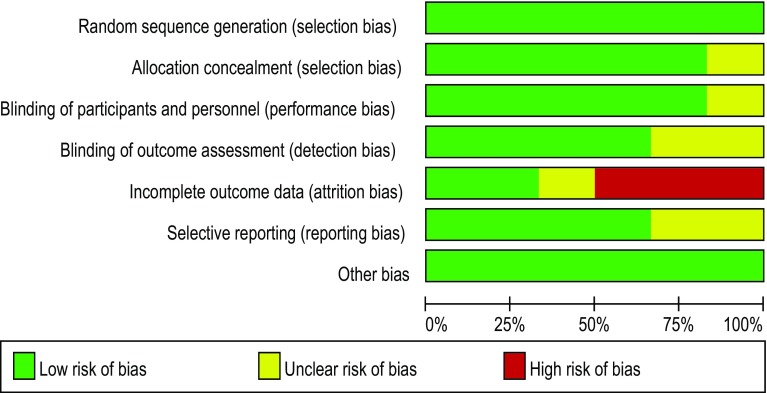
Risk of bias for studies included in this meta-analysis

**Fig. 3:**
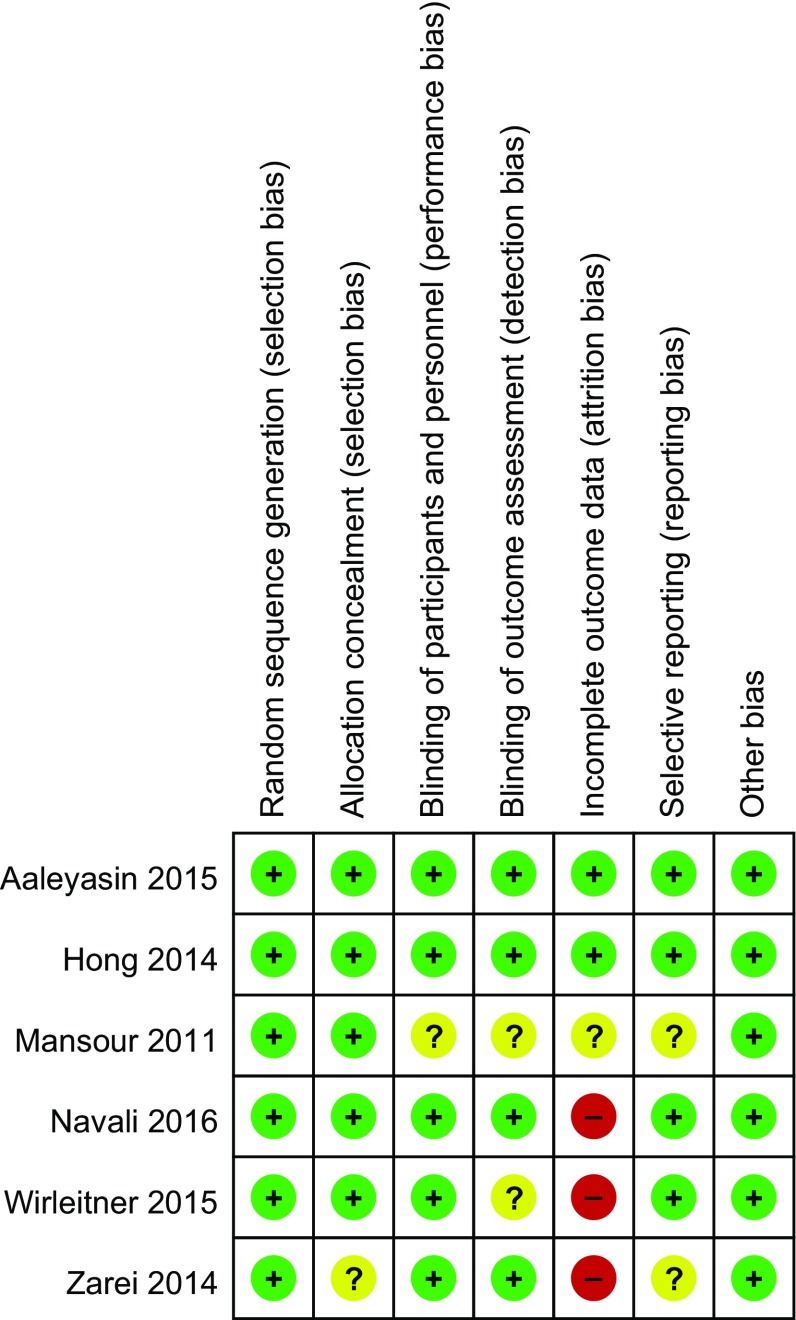
Risk of bias for studies included in this meta-analysis

The 6 eligible studies enrolled a total of 2759 participants (hCG group: *n* = 1429; control group: *n* = 1330). The characteristics of the trials (see Table 1) included the author, inclusion criteria, sample size, type of cycle, treatment protocol, dose, embryo stage, type and timing of intrauterine hCG, and reports of all outcomes. The studies’ risks for bias are shown in Figs. 2 and 3.

**Table 1:** Characteristics of the studies included in the meta-analysis

	Country	*N*		Patients’ age (years)	Treatment		Embryo stage	Outcome (rates)
		hCG group	Control group		hCG group	Control group		
Mansour et al. [3]	Egypt	100 IU hCG: 83 200 IU hCG: 84 500 IU hCG: 107	Control 1: 93 Control 2: 105	≤ 40	100, 200, and 500 IU hCG in 0.04 ml culture medium 7 min before ET	No hCG or culture medium	Day 2 or 3, cleavage stage	Clinical pregnancy, implantation, miscarriage, delivery
Hong et al. [14]	New Jersey	148	1152	< 43	500 IU hCG (purified urinary) in 0.02 ml culture media less than 3 min before ET	0.02 ml culture media without hCG	Day 6, fresh or frozen, blastocyst stage	Sustained implantation, ongoing pregnancy
Zarei et al. [15]	Iran	84	98	18–40	rhCG 250 μg (0.5 ml), 12 min before ET	0.5 ml normal saline instead of hCG, 12 min before ET	Day 3, cleavage stage	Implantation, clinical pregnancy, ongoing pregnancy, abortion and ectopic pregnancy
Aaleyasin et al. [16]	Iran	240	243	< 40	500 IU hCG (urinary) in 0.05 ml culture media 5–7 min before ET	0.05 ml culture media without hCG	Day 2 or 3, cleavage stage	Implantation, clinical pregnancy, spontaneous, abortion, live birth rate
Wirleitner et al. [5]	Austria	Cohort A: 89 Cohort B: 510	Cohort A: 93 Cohort B: 494	≤ 43	500 IU hCG in 0.04 ml of culture media, cohort A infusion on Day 3 after ovum pick-up; cohort B infusion 3 min before ET	0.04 ml culture medium without hCG	Day 5, blastocyst, SET or DET	Pregnancy, clinical pregnancy, implantation, live birth rate, miscarriage
Navali et al. [8]	Iran	71	67	≤ 41	500 IU: 0.1 ml (500 IU hCG) and 0.4 ml normal saline were given via insulin syringe immediately after oocyte retrieval	0.5 ml normal saline instead of hCG	Day 3	Biochemical pregnancy, clinical pregnancy, implantation, ongoing pregnancy, abortion, ectopic pregnancy

*hCG* human chorionic gonadotropin, *rhCG* recombinant human chorionic gonadotropin, *ET* embryo transfer, *SET* single embryo transfer, *DET* double embryo transfer

### Implantation rate

**Fig. 4:**
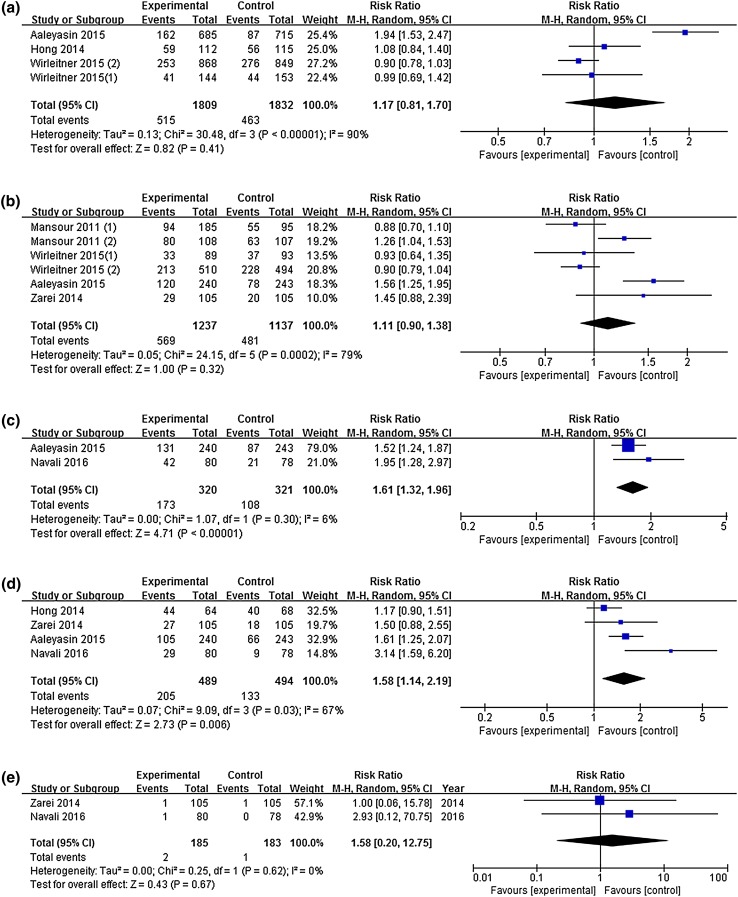

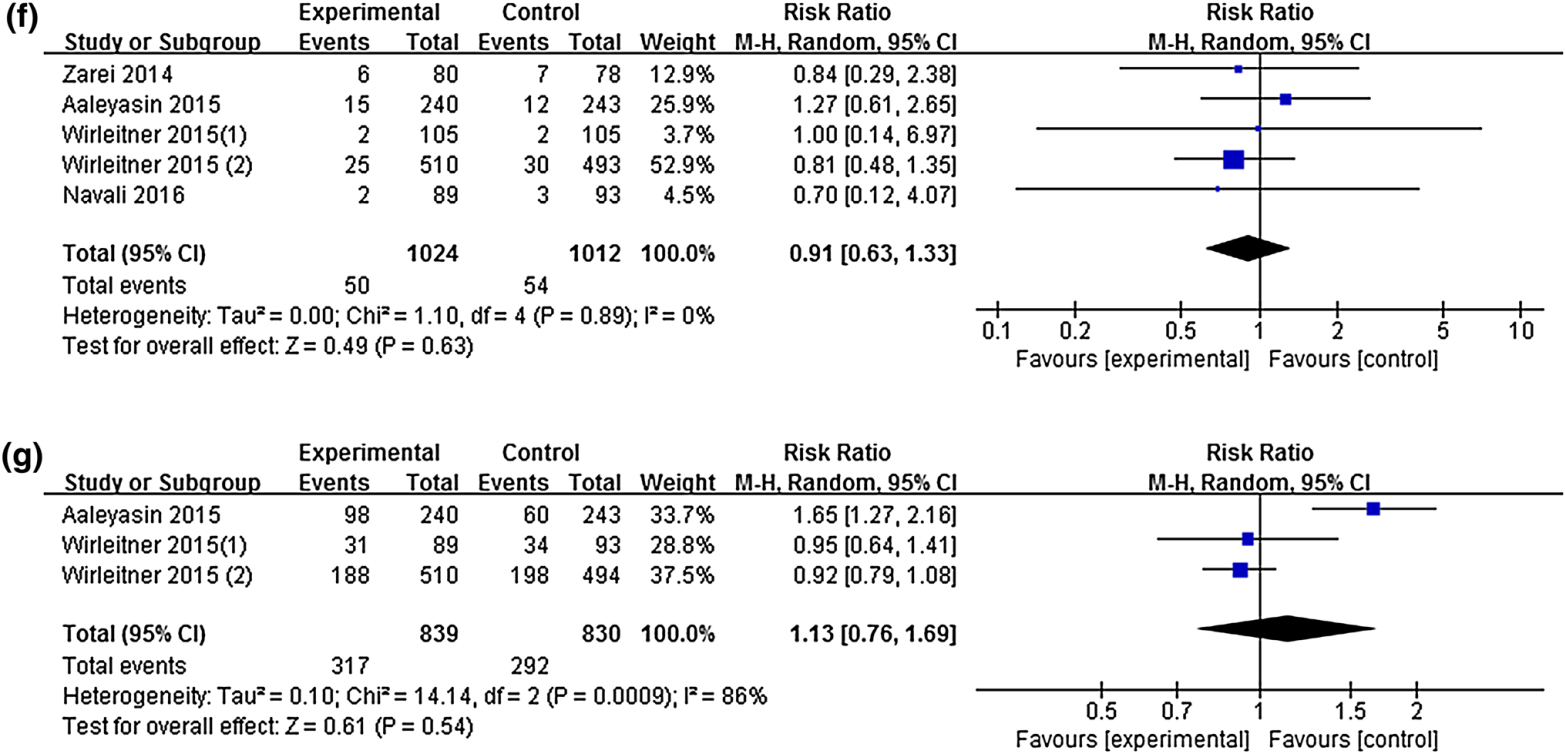
Forest plot: **a** implantation rate. **b** Clinical pregnancy rate. **c** Biochemical pregnancy rate. **d** Ongoing pregnancy rate. **e** Ectopic pregnancy rate. **f** Miscarriage rate. **g** Live birth rate for intrauterine hCG administration versus no hCG before fresh ET

Three studies with four experimental arms reported clinical pregnancy rates [5, 14, 16]. Pooling the results of the three studies (*n* = 3641) showed no significant differences between the two groups in implantation rates [RR 1.17, 95% CI (0.81, 1.70), *P* = 0.41]. Significant heterogeneity between the studies on implantation rate was found (*I*^2^ = 90%; *P*_heterogeneity_ < 0.00001) (Fig. 4a).

### Clinical pregnancy rate

Four studies with six experimental arms reported clinical pregnancy rates [3, 5, 15, 16]. Their pooled results (*n* = 2374) revealed no significant difference in the clinical pregnancy rate between the two groups [RR 1.11, 95% CI (0.90, 1.38), *P* = 0.32]. There was significant heterogeneity between the studies in clinical pregnancy rates (*I*^2^ = 79%; *P*_heterogeneity_ = 0.0002) (Fig. 4b).

### Biochemical pregnancy rate

Biochemical pregnancy rates were reported in two studies [8, 16] and the rate was significantly higher in the hCG group [RR 1.61, 95% CI (1.32, 1.96), *P* < 0.00001]. No significant heterogeneity was found between the studies (*I*^2^ = 6%; *P*_heterogeneity_ = 0.30) (Fig. 4c).

### Ongoing pregnancy rate

Ongoing pregnancy rates were reported in four studies [8, 14–16] and the rate was significantly higher in the hCG group [RR 1.58, 95% CI (1.14, 2.19), *I*^2^ = 67%, *P *= 0.006]. There was significant heterogeneity between the studies in ongoing pregnancy rates (*I*^2^ = 67%; *P*_heterogeneity_ = 0.03) (Fig. 4d).

### Ectopic pregnancy rate

Ectopic pregnancy rates were reported in two studies [8, 15]. No significant difference in ectopic pregnancy rates was found between the two groups [RR 1.58, 95% CI (0.20, 12.75), *P *= 0.67] and no significant heterogeneity was found between the studies (*I*^2^ = 0%; *P*_heterogeneity_ = 0.62) (Fig. 4e).

### Miscarriage rate

Miscarriage rates were reported in four studies with five experimental arms [5, 8, 15, 16]. No significant difference in miscarriage rates was found between the two groups [RR 0.91, 95% CI (0.63, 1.33), *P *= 0.63] and no significant heterogeneity was found between the studies (*I*^2^ = 0%; *P*_heterogeneity_ = 0.89) (Fig. 4f).

### Live birth rate

Live birth rates were reported in two studies with three experimental arms [5, 16]. No significant difference in the live birth rates was found between the two groups [RR 1.13, 95% CI (0.76, 1.69), *P *= 0.54]. There was significant heterogeneity in live birth rates between the studies (*I*^2^ = 86%; *P*_heterogeneity_ = 0.0009) (Fig. 4g).

### Sensitivity analysis and publication bias

Five of the six RCTs had scores of four or higher on the Jadad scale and were, therefore, included in the sensitivity analysis. No significant changes were found in any of the outcomes, except for biochemical pregnancy and ongoing pregnancy, which were found to be significantly higher in the hCG group than the control group.

We did not construct funnel plots to examine publication bias, or perform meta-regression analyses or subgroup analyses because of the small number of RCTs included in this meta-analysis.

## Discussion

This meta-analysis with 2759 patients from 6 RCTs examined the effect of intrauterine injection of hCG before fresh ET on pregnancy outcomes, and showed that this intervention did not improve the live birth rate for the fresh ET cycle.

It has recently been suggested that a freeze-all strategy may improve IVF outcomes [21]. Better IVF outcomes using this strategy are partly attributed to being disengaged from ovarian stimulation in the ovarian stimulation cycle, which can have a negative effect on the receptivity of the endometrium for embryo implantation. A retrospective cohort study that included 20,687 women who started their first IVF cycle using the freeze-all strategy reported that the live birth rate was 50.74% after the first complete cycle, which was higher than the live birth rate using the conventional IVF strategy [22]. Alison’s study found the ongoing pregnancy rate and live birth rate were significantly higher in the freeze-all group compared with the fresh group when known euploid embryos were transferred [23]. As the transfer of frozen embryos yields a higher pregnancy rate, our focus was on how to improve pregnancy outcomes for the fresh cycle.

The crucial role of hCG in the maintenance of pregnancy has been well documented; it can improve endometrial receptivity, promote embryo–endometrial cross talk [3, 11, 12, 20], and support the maternal corpus luteum in early pregnancy [24]. Beneficial effects of intrauterine hCG injection before ET on outcomes of IVF/ICSI cycles have been reported by many authors [8, 15]. The first study by Mansour et al. [3] found that intrauterine hCG injection before ET improved IVF/ICSI outcomes, with the pregnancy rate of the 500 IU hCG-group significantly higher than the control group’s rate. A later study by Navali et al. [8] also found that an intrauterine hCG injection group had higher rates of implantation, clinical pregnancy, and ongoing pregnancy. It is likely that the intrauterine administration of hCG before ET causes numerous changes in the endometrium, as described in the experiments conducted by Licht et al. [11] and Mansour et al. [3]. A meta-analysis published in 2015 reported similar results [25].

However, after analyzing eight RCTs, Osman et al. [26] concluded that the current evidence did not support the use of intrauterine hCG injection before ET. A total of 3087 women undergoing IVF/ICSI (intrauterine hCG group: *n* = 1614; control group: *n* = 1473) were enrolled. No significant differences were found in the live birth rate or spontaneous abortion rate between the intrauterine hCG group and the control group. It should be noted that they did not discuss fresh or frozen ET separately. They speculated that the acquisition of physiological effects of hCG required certain physiological doses in a timely fashion, which should take into account the stages of embryonic development and endometrial receptivity. Three isoforms of hCG (hyperglycosylated hCG, hCG, and beta hCG) are produced by the embryo, cytotrophoblast, and syncytiotrophoblast. Their dominance levels differ depending on the stage of the embryo and the pregnancy [27, 28]. Different stages require different isoforms, and changes in isoforms might not be achieved by injecting high doses of hCG.

A retrospective case–control study conducted at a multi-site private IVF clinic with 34,259 ETs of which 656 received intrauterine hCG infusions, reported that intrauterine hCG injection before ET not only seemed to have no benefit, but had a negative effect on fresh ETs [29]. Intrauterine hCG administration in fresh ETs was associated with a lower clinical-pregnancy rate and a downward trend in the live birth rate. These findings raise the new hypothesis that increasing quantities of hCG might interfere with endometrial receptivity.

Our results suggest that the use of intrauterine hCG injection before fresh ET does not improve IVF/ICSI outcomes. There were no significant differences in implantation, clinical pregnancy, or live birth rates between the intrauterine hCG injection group and the control group. Several factors may contribute to these results. The dosages and types of hCG varied among the six RCTs. A meta-analysis published in 2016 found that a hCG dose of 500 IU or greater showed promise as the dosage before cleavage-stage ETs, but a hCG dose less than 500 IU before cleavage-stage ETs had no effect, and a dose of 500 IU or greater before blastocyst-stage ETs also showed no benefit [30]. Mansour’s study [3] reported that a 100- or 200 IU-hCG injection did not increase the pregnancy rate. However, a 500 IU-hCG injection before ET significantly improved the pregnancy rate. Furthermore, the stages and numbers of embryos transferred were different in the studies.

The schedules for hCG injections were different among the 6 RCTs, and the researchers’ lack of explanations for the chosen times was confusing. The timing of hCG infusions were 3 min; less than 3 min; 7 min; 5–7 min; 12 min, and immediately following oocyte retrieval. Whether hCG’s effect on the endometrium and embryos was transient remains unclear. The contractions of the uterine cavity during the transplantation process is also a matter of concern. Chung’s study [31] found that the live birth rate was significantly reduced in those with a higher frequency of uterine contractions 5 min after ET (by 60 min after ET the frequency of uterine contractions returned to the baseline). Therefore, the time of hCG infusion might be an important variable affecting clinical outcomes.

In our study, there were no significant differences in clinical pregnancy, implantation, miscarriage, ectopic, or live birth rate between the hCG group and control group. However, the biochemical pregnancy rate in the study group was significantly higher than in the control group. An important reason for this result is that the analysis consisted of only two articles although biochemical pregnancy rate was defined as a β-hCG rise 14 days after ET in both studies. The ongoing pregnancy rate was also an unexpected outcome. We included four articles that showed a higher ongoing pregnancy rate in the experimental group. There are two possible explanations for this result. First, Aaleyasin’s study [16] had a large sample size, which might have biased the overall results of the analysis. Second, this result might be related to the use of different endpoints in the studies. Ongoing pregnancy was defined as a pregnancy longer than 14 weeks in Navali’s study, but it was defined as a pregnancy longer than 20 weeks in other studies. Pregnant women are still at risk for miscarriage between 14 and 20 weeks.

It is worth mentioning that the clinical pregnancy rate [RR 1.11, 95% CI (0.90, 1.38)] and the ongoing pregnancy rate [RR 1.58, 95% CI (1.14, 2.19)] in the experimental group showed an increasing trend. Similarly, the miscarriage rate [RR 0.91, 95% CI (0.63, 1.32)] was still on the rise. Our study analyzed only six RCTs. If the number of studies is increased, the results might be different; therefore, further observations are needed.

To the best of our knowledge, this meta-analysis is the first to evaluate the effect of intrauterine hCG injection at the time of fresh ET. The heterogeneity of several of the comparisons was greater than 50%; therefore, we explored the sources of heterogeneity and found three key variables which made sense: embryo-stage hCG dose, and timing of the intrauterine hCG injection. We did not conduct subgroup analyses due to the limited sample size. Only two RCTs reported live birth rates; therefore, the results of the current study should be interpreted with caution.

## Conclusion

The current evidence for intrauterine injection of hCG before fresh ET does not support its use in ET cycles. Well-designed studies and well-conduced multicenter trials are needed in the future.
